# A Potential Role for Photobiomodulation Therapy in Disease Treatment and Prevention in the Era of COVID-19

**DOI:** 10.14336/AD.2020.0901

**Published:** 2020-12-01

**Authors:** Ann Liebert, Brian Bicknell, Wayne Markman, Hosen Kiat

**Affiliations:** ^1^Faculty of Medicine and Health, University of Sydney, Sydney, Australia.; ^2^Research and Governance, Adventist Hospital Group, Wahroonga, Australia.; ^3^SYMBYX Pty Ltd, Artarmon, Australia.; ^4^Faculty of Health Science, Australian Catholic University, North Sydney, Australia.; ^5^School of Business, University of Technology, Sydney, Australia.; ^6^Cardiac Health Institute, Sydney, Australia.; ^7^Faculty of Medicine, University of NSW, Kensington, Australia.; ^8^Faculty of Medicine, health and Human Sciences, Macquarie University, Macquarie Park, Australia

**Keywords:** COVID-19, photobiomodulation, immunomodulation, mitochondrial dysfunction, microbiome

## Abstract

COVID-19 is an evolving pandemic that has far reaching global effects, with a combination of factors that makes the virus difficult to contain. The symptoms of infection can be devastating or at the least very debilitating for vulnerable individuals. It is clear that the elderly are at most risk of the adverse impacts of the virus, including hospitalization and death. Others at risk are those with comorbidities such as cardiovascular disease and metabolic conditions and those with a hyper-excitable immune response. Treatment options for those with acute responses to the virus are limited and there is an urgent need for potential strategies that can mitigate these severe effects. One potential avenue for treatment that has not been explored is the microbiome gut/lung axis. In addition to those severely affected by their acute reaction to the virus, there is also a need for treatment options for those that are slow to recover from the effects of the infection and also those who have been adversely affected by the measures put in place to arrest the spread of the virus. One potential treatment option is photobiomodulation (PBM) therapy. PBM has been shown over many years to be a safe, effective, non-invasive and easily deployed adjunctive treatment option for inflammatory conditions, pain, tissue healing and cellular energy. We have also recently demonstrated the effectiveness of PBM to alter the gut microbiome. PBM therapy is worthy of consideration as a potential treatment for those most vulnerable to COVID-19, such as the elderly and those with comorbidities. The treatment may potentially be advantageous for those infected with the virus, those who have a slow recovery from the effects of the virus and those who have been denied their normal exercise/rehabilitation programs due to the isolation restrictions that have been imposed to control the COVID-19 pandemic.

First reported in December 2019 (www.who.int/csr/don/05-january-2020-pneumonia-of-unkown-cause-china/en/), more than 21 million people have now been infected by the new Severe Acute Respiratory Syndrome Coronavirus 2 (SARS-CoV-2). The infection per se, and its therapy, have claimed over half-a million deaths worldwide, which continues to rise daily www.worldometers.info/coronavirus/.

There are dozens if not hundreds of different types of coronaviruses, four of which have been shown to cause mild and mainly upper respiratory infections (the so called common cold); 2 strains cause severe and frequently lethal outbreaks of respiratory infections, one in 2002 referred to as SARs (severe acute respiratory syndrome), the other, MERs (Middle East Respiratory Syndrome) occurred in 2012. The third and latest coronavirus pandemic, raging since late 2019 is caused by yet another strain. The virus particle is around 100 nanometres in diameter and can be seen only through an electron microscope. The aerosol or droplet transmission of the virus to humans gains entry through mucosal surfaces of the mouth, nose and eyes [1]. Viral entry is mediated through the angiotensin converting enzyme 2 (ACE2) receptor, a membrane-associated enzyme expressed in vascular endothelia, renal and cardiovascular tissue as well as epithelia of the small intestine. The relationship between ACE2 expression and infection has not been determined [2]. The treatment of cardiovascular disease (CVD) with angiotensin-converting enzyme inhibitors and angiotensin receptor blockers have the additional effect to upregulate ACE2 expression, which may play a significant role in individual viral responses.

The median incubation period before symptoms might become evident is 4.9 - 5.8 days, with a range of 1 - 14 days [3]. Once infected and thus contagious, the relatively long incubation period in humans facilitates the spread of the infection [3]. Beyond the incubation period, symptoms of the infected vary widely, from none or minimal symptoms, to florid and rapidly progressive respiratory distress. The elderly are the most predisposed to the deleterious sequalae of COVID-19 [4-6], most probably due to an aging immune system, increased manifestations of inflammatory conditions and accumulated mitochondrial dysfunction [7]. In the USA, 77% of all deaths are in the over 65 years age group [5]. Those with chronic comorbidities that impact their immune system are the second group at risk, including CVD [8], type II diabetes (T2D) [9, 10], chronic respiratory disease [10], hypertension [10], cancer [11], metabolic syndrome [12] and obesity [13, 14]. It has been reported that less than 1% of all deaths from COVID-19 do not have a comorbidity [15]. A third susceptible group are those with a hyper-excitable neuro-immune axis, which affects the nervous system as well as endothelial and vascular responses leading to an over-intense immune response. These people are often younger and can have hyper-excitable physiological responses to environmental stressors. Risk factors for this response include genotypes for ion channelopathies such as migraine, erythromyalgia and epilepsy, resulting in these endothelial susceptibilities [16]. This group can be of higher intelligence [17] and are possibly over-represented in health care workers. Health care workers possibly have a higher morbidity and mortality compared to the general population [18], which may be influenced in some way by this immune hyper-excitability.

Those susceptible to the virus may succumb to dysregulated immunity, recalcitrant deoxygenation and respiratory distress, multi-organ failures and debility resulting from prolonged a catabolic state. Importantly, they also often suffer adverse reactions (ADRs) to therapeutic interventions including the established and potential cardiovascular ADRs related to the antibiotics and high dose corticosteroids, anti-viral and immune modulating drugs currently used in the management of COVID-19 infections [19, 20]. Other features of infection include cardiovascular injury [2], atrial fibrillation [21], central and peripheral nervous system symptoms [22], which affect over a third of the infected patients [23], mouth ulcers and hyposmia/anosmia (loss of smell). Rapid and fulminant progression to pneumonia can occur in the elderly and people with comorbidities. This is thought to occur as a result of an initial poor immune response followed by an inappropriate hyper-immune reaction or “cytokine storm”. The resulting unrelenting inflammation affects vital organs including pulmonary tissue and vascular structures [6]. This over-responsive immune response may be associated with neutrophil recruitment and activity [24]. The subsequent cellular damage and multi-organ failures heighten mortality risk more so than the infection itself and is particularly prevalent among the aged and those with comorbidities. Immunosuppressive therapies including corticosteroids https://clinicaltrials.gov/ct2/show/NCT04355247 to prevent or placate the cytokine storm [25, 26] in COVID-19 have so far only met with modest success. More targeted therapies are urgently needed.

Recovery from COVID-19 is frequently protracted and the long-term prognosis remains to be fully realized. An unknown percentage will have ongoing symptoms after discharge from hospital and/or recovery from overt respiratory and other life-threatening symptoms. It has been reported www.theguardian.com/australia-news/2020/jul/17/most-covid-19-patients-admitted-to-a-sydney-hospital-in-march-still-have-symptoms that at 3- or 4-months post hospital discharge, up to 80% of recovered COVID-19 patients continued to be symptomatic. Nonspecific aches and pain, dyspnoea, palpitations, joint and chest pain, dizziness or light-headedness, headaches, fatigability, hyposmia and anosmia are not infrequent [27]. More serious ongoing symptoms can include pulmonary hypertension and interstitial fibrosis, pericardial effusion and myocarditis, neurologic and neuropsychiatric sequalae including dysautonomia [28], myalgic encephalomyelitis/chronic fatigue syndrome (ME/CFS) [29], depression [30] and autoimmune disease [31].

A less recognised consequence of the COVID-19 pandemic is the unintended effect that the measures taken to contain the pandemic have had on vulnerable individuals. Many of these individuals have been unable to socialise or to attend exercise and rehabilitation classes due to the lockdown period and the social isolation necessary to combat spread of the virus. The stress of this can have the effect of reducing the resilience and immunity of the very individuals (the elderly and those with comorbidities) that the measures are designed to protect [32], thus increasing the chances of an unfavourable outcome if infected.

A magic bullet solution for the current COVID-19 pandemic is unlikely in the near future, although much research is directed towards an effective vaccine. A wide range of social and lifestyle measures to reduce cross infection have been met with success in some but not other regions or countries [33]. Development and discovery of novel and effective pharmacotherapy, vaccines for disease prevention and repurposing existing drugs to fight the COVID-19 are being robustly pursued [34] but there is also a need for broad scale clinical trials of potential strategies, including interdisciplinary collaborations, aimed at mitigating the severe effects and side-effects of the COVID-19 pandemic [5].

## The link to the gastrointestinal microbiome

There is a strong link between the gut microbiome and susceptibility to disease and most likely to COVID-19 [35]. The gut microbiota is well known to affect immunity [36, 37], interacting with the gut mucosa and stimulating the production of both pro and anti-inflammatory cytokines. Low inflammatory conditions support a healthy gut microbiota, which in turn contributes to maintaining non-inflammatory conditions. A cascade into dysbiosis leads to a disruption of the mucosal barrier, allowing microbial products of the dysregulated microbial population to leak into surrounding tissues and increase the inflammatory response, which further increases dysbiosis. This contributes to generally reduced immunity and to the comorbidities known to contribute to COVID-19 susceptibility (such as obesity, T2D, heart disease). Elderly individuals frequently suffer a decrease in microbial diversity in the gut, contributing to dysbiosis. In addition, there is a gut/lung axis that links the microbiome of the gut with lung health [38], with gut metabolites transferred to the lung [37] and the gut bacteria playing an important role in the response to acute respiratory distress syndrome (ARDS) [35]. Early results of the microbiome analysis of a small number of hospitalized COVID-19 patients indicated that the microbiome was adversely impacted, with depletion of bacteria representative of a healthy microbiome and enrichment of opportunistic pathogens [39]. These changes were correlated with disease severity and persisted throughout the hospitalization period.

Changing the composition of the gut microbiota with diet and supplements can improve immunity generally [40], while altering the microbiome (with soluble fibre) has been shown to reduce the severity of allergic lung inflammation [41]. Modulation of the gut microbiome (with diet, soluble fibre, probiotics) has been suggested as a potential way to assist viral respiratory infections generally [42] and SARS-Cov-2 in particular [35]. There has been speculation that the gut microbiota can have an influence on ACE2 receptors and cardiovascular health and are therefore a potential target for cardiopulmonary therapy [43]. Additionally, the ACE2 receptors are also expressed in the gut enterocytes [35]. The expression of ACE2 (in a mouse model) can be regulated by certain species of the gut microbiome (*Bacteroides* species), part of the population depleted during COVID-19 hospitalization [39]. The implications of this has yet to be investigated.

The oral microbiome is also an important component of immunity. A healthy microbial population can be disturbed by a dysregulated immune system, an inflammatory response or poor oral hygiene [44]. A disturbed microbiota is implicated in a number of diseases, such as periodontitis, CVD and Alzheimer’s disease [45] as well as being less able to prevent viral infection [46]. Interestingly, periodontal disease has a strong association with obesity, CVD, T2D as well as aging, the same comorbidities associated with a poor prognosis with COVID-19 and the potential of oral microbiome dysbiosis and susceptibility to COVID-19 has been raised [47, 48]. Improvement in oral hygiene is suggested as a way to maintain a healthy oral microbiome, which may be somewhat protective against viral infection [49].

## Photobiomodulation (PBM) therapy in clinical medicine

Throughout the ages, light, including sunlight has been known for its wide-ranging health effects for myriad maladies. In 1903 Dr. Niels Ryberg Finsen, a Danish physician was awarded the Nobel Prize in Physiology or Medicine for his work in treating tuberculosis with ultraviolet or blue light and smallpox with red light [50]. The contemporary clinical practice of PBM therapy, often also referred to as low-level laser therapy, is the result of on-going evolution since its first application over half-a century ago, when the work of Dr. Endre Mester and colleagues at the Semmelweis Medical University in Hungary demonstrated its therapeutic benefits for wound healing [51].

PBM is the use of narrow wavelength bands of light (either LED or laser) to modulate cellular responses with no thermal effect. Putatively, PBM carries no risk to health [52-54], its safety profile equating that of ultrasound tests. Unlike much pharmaceutical therapy, PBM is free of serious deleterious side-effects and is, by its nature, non-invasive. PBM therapy is mostly delivered for no more than 10-20 minutes through portable, handheld or wearable devices and is safely repeatable. Measurable symptomatic and clinical benefits can result from a single treatment but PBM therapy is usually provided as a course of several treatment sessions.

The main target of PBM is considered to be the electron transport chain of the mitochondria, in particular complex IV, cytochrome-C-oxidase, which acts as a chromophore, absorbing red and near infrared light [55]. The effect of this absorption is thought to be the release of reactive oxygen species (ROS) from the complex, allowing increased membrane potential, increased ATP production and downstream cellular signalling via ATP, cAMP, ROS, Ca^2+^ and nitric oxide (NO) to influence gene transcription [55]. There are also many other chromophores capable of absorbing light with resulting physiological effects, such as opsins and light-activated ion channels. The most effective wavelength for delivery of PBM in immune modulation is likely to be in the red and near-infrared range, based on the cytochrome-C-oxidase and porphyrin absorption peaks being centred at 640 nm and HbO_2_ at 900nm [55]. The energy required for effective PBM is low, in the range of 1 to 16 joules/cm^2^. The PBM dose is biphasic, meaning that above a certain threshold (outside of the dose window) increasing the energy will not increase the therapeutic effect [56].

**Figure 1.**
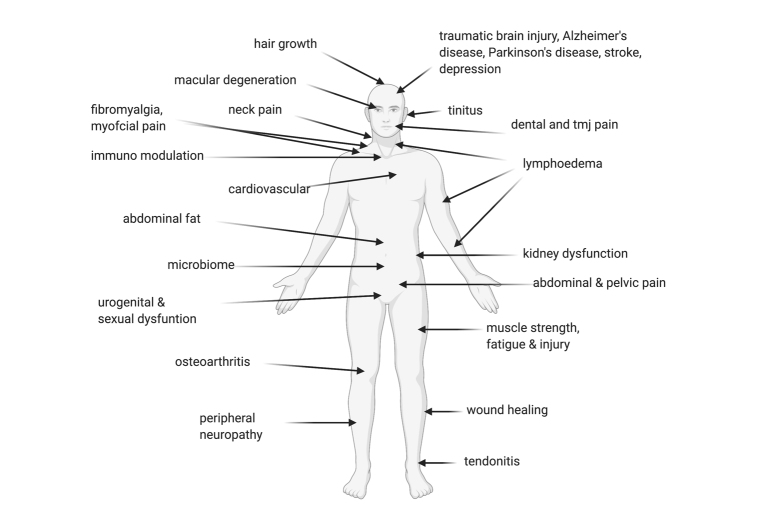
Conditions that have been shown to be successfully treated using photobiomodulation therapy.

PBM has a multitude of effects on the body, in many organ systems and is able to treat various disorders (Figure 1), through its action at the cellular and mitochondrial level [55]. In experimental models, the degree and type of immune responses to PBM are influenced by the anatomic surface where treatment is applied. For example, immunomodulatory effects appeared to be more effective when applied on the thymus area compared to limbs, with a favourable rise in interleukin (IL)-2, NO and heat shock protein 70 production. Treatment dose, cumulative dose and duration of exposure also appear to pay a role where unduly prolonged treatment duration may even cause attenuation and reversal of treatment efficacy toward immunosuppression [57].

The literature is replete with experimental and clinical trials demonstrating therapeutic efficacy of PBM in a multitude of disease process, including inflammation. PBM therapy has recently been recommended as standard care for the treatment of oral mucositis following chemo or radiation therapy in the MSCC/ISOO guidelines www.mascc.org/mucositis-guidelines.

## Photobiomodulation therapy and general health

PBM has been shown to improve the general health and resilience of cells and tissues. The effect of PBM to improve mitochondrial metabolism and ATP generation leads to increased muscle strength and performance in sports and athletics [58, 59] and to reduced muscle wasting and degeneration in animal and cell culture models [60-62]. This makes it a candidate for treatment of COVID-19 cases under respiratory distress. PBM has been demonstrated to be more effective in damaged or diseased cells, tissues and individuals [55]. PBM has also been shown to have an effect in chronic obstructive airway disease when the muscles of the chest wall are treated [63]. Efficient mitochondria are also important in overcoming disease and in the recovery process. Often, mitochondrial dysfunction increases with age and may not be sufficient to enable recovery after infective disease and other immune insults [64]. PBM is known to enhance mitochondrial function, but the positive effect of PBM on the aging process in animal models has yet to be demonstrated in humans [65]. The action of PBM on the mitochondria also has the effect of activating transcription factors, which can lead to increased expression of genes involved in inflammatory signalling [66].

PBM is effective at reducing myocardial infarct size and reducing inflammation in animal models [67], has been suggested as therapy in human CVD [68] and has been shown to modulate the expression of ACE2 [67]. PBM has also been shown to improve blood flow and oxygenation [69, 70], both peripherally and in the CNS most probably due to the release of NO, an important vasodilator. PBM therapy has been used to treat post-viral and chronic fatigue, as well as fibromyalgia and other instances of centrally mediated pain [71].

## Evidence and potential mechanisms of PBM in immunomodulation

PBM appears to exert pluripotent effects in the modulation of inflammation and immunity [72]. Many studies have demonstrated that PBM modulates inflammation by reducing the pro-inflammatory cytokines (such as IL-1β, IL-6, IL-8, TNF-α) and other inflammatory markers released from activated inflammatory cells, while increasing the anti-inflammatory cytokines (IL-10) [72]. The immuno-modulatory effect of PBM on cytokines regulation and the complement cascade occurs via the POMC pathway, involving regulation of the hypothalamic pituitary axis through the direct modulation of the POMC/melanocortin signalling pathway including a-MSH, a potent anti-inflammatory molecule. The POMC pathway is regulated by PBM [73], which in turn modulates both ACTH and β-opioid, as well as, interestingly, ACE activity [74].

One of the central effects of PBM on the immune response is via the modulation of neutrophil function [75] by balancing neutrophil numbers, improving neutrophil efficiency and modulating the neutrophil extracellular trap formation [76]. Reducing over-accumulation of neutrophils is a major mechanism for the effect of PBM in reducing acute lung inflammation [77]. This is crucial in preventing the cytokine storm cascade in autoimmune diseases. PBM also modulates the ratio of M1 and M2 macrophage phenotypes, reducing pro-inflammatory cytokines and chemokines and increasing anti-inflammatory cytokines and thus balance the inflammatory process [78].

These inflammatory changes facilitated by PBM have profound effects on many body processes. For example, PBM therapy has been shown to modulate peripheral blood mononuclear cells and CD4+ cells to reduce inflammatory effects in multiple sclerosis patients and healthy adults by increasing IL-10 and reducing IFN-γ [79, 80]. PBM reduces the number of inflammatory cells, pro-inflammatory cytokines as well as fibrotic tissue in a mouse model of lung fibrosis [81]. Acute lung inflammation in rats is reduced with PBM to reduce oedema, neutrophil influx and TNF-α, while reducing IL-10 in rats [82].

In an experimental model of induced acute peritonitis in rats, Yu and co-workers [83] showed PBM resulted in lymphocyte proliferation and enhanced lymphocyte ATP synthesis compared to controls, and the 60-day survival rate of the PBM group was double that of the control group (p<0.001). Assis et al [84] further demonstrated the immune modulation capability of PBM, with septic rats treated with PBM exhibiting lower IL-6 activity and decreased atrogin-1 and MuRF-1 immuno-expression (markers of sepsis related muscle catabolic states).

PBM causes mitogenic stimulation responsive lymphocyte proliferation and enhanced lymphocyte ATP synthesis [83]. A plausible mechanism for PBM induced lymphocytic proliferation is through the reaction of light with haemoglobin, resulting in oxygen radical production [85]. Indeed, in immunological cells, PBM induces production of reactive oxygen species, NO or interleukins most often, leading to an anti-inflammatory effect [85]. It is well documented that various immune response processes are highly dependent on cellular energy, the latter being depressed in sepsis and septic shock cases [86, 87]. The mitochondria probably act as photo-acceptors for PBM and robustly reactivate cellular energy synthesis to re-establish ATP levels in a variety of cells including lymphocytes and macrophages, and through several pathways that trigger activation of nucleic acid synthesis and cellular proliferation [88, 89].

## PBM in airway inflammation, gut microbiome and dysautonomia

PBM has been shown to be effective in controlling neutrophil activation, thus restoring the balance between pro and antioxidant mediators by reducing pro-inflammatory cytokines (IL-6, TNF-α) and increasing anti-inflammatory cytokines (IL-10) in a mouse model of acute lung injury induced by gut ischemia and reperfusion [82, 90]. This has also been shown in mouse models of pulmonary fibrosis [91] and chronic obstructive airway disease induced by tobacco smoke [92]. The infiltration of neutrophils into the lungs, which contributes to inflammation, is also reduced by PBM [78].

The efficacy of PBM therapy to treat pneumonia has been reported in 48 infants treated with conventional therapy who also received laser therapy with “Vostok” laser therapeutic devices for 2-3 days, compared to 45 infants receiving conventional therapy alone and another 18 healthy newborns as controls. In a trial of using red light therapy to treat retinopathy of prematurity [93], one notable side-effect was the survival of all 21 premature infants in the treatment group, while 4 infants in the non-treatment group died from lung complications (pers. com. Prof Krisztina Valter). It has also been reported that ARDS can be successfully treated with PBM therapy [94].

We have previously shown [95] that PBM can alter the gut microbiome in a favourable way in a mouse model. We have also demonstrated favourable changes in the gut microbiome in a number of human trials (manuscript in preparation) and are currently investigating the potential of PBM to alter the oral microbiome. One potential mechanism for the effect on gut microbiota is the reduction of inflammation in the adipose tissue of the abdomen afforded by PBM. Improving the gut microbiome from a dysbiotic state, whether by diet, prebiotics, exercise or PBM, will reduce inflammatory processes, improve general health and protect against future immunological insults [96], including, perhaps, a future cytokine storm.

Recently there has been much interest in the use of transcranial PBM to address many symptoms of neurological and neuropsychiatric disorders [97]. Transcranial devices have been shown to modulate neural oscillations [70, 98], improve cognition in healthy adults, improve cognitive performance of people with TBIs [99] and improve symptoms of depression [100]. We have demonstrated a positive effect of PBM therapy in improvement of cognition scores in individuals with Parkinson’s disease (manuscript in preparation).

## The potential of PBM for COVID-19

A number of recent publications have suggested that PBM therapy may be of benefit in the treatment and/or recovery of COVID-19 [101, 102] by targeting the blood, either directly or trans-dermally [102, 103] and/or targeting the lungs [104]. At least one trial of PBM therapy to the respiratory muscles is underway https://clinicaltrials.gov/ct2/show/NCT04386694 and PBM therapy is being trialled as a therapy for COVID-19 in Russia www.lazmik.ru/assets/templates/docs/Coronaviridae_SARS_COVID-19_LLLT_protocol_eng1.pdf. There has been one recent case report of the effectiveness of PBM therapy to treat a patient with severe COVID-19 pneumonia [105].

There are a number of areas in the COVID-19 crisis that may benefit from PBM therapy, especially among the elderly and other individuals with comorbidities or conditions that make them especially vulnerable to the virus:

1. Individuals infected with the virus and who are admitted to intensive care units may benefit from PBM therapy to the chest to help improve airways, improve blood oxygenation and increase muscle performance to assist with breathing. PBM may also help to balance the immune system and reduce immune hyperactivity to resist progression to a cytokine storm. The same mechanisms may help vulnerable individuals infected with the virus to avoid the worsening of symptoms that would otherwise lead to admission to hospital.
2. The main clinical benefit of PBM therapy in COVID-19, however, appears to be for patients who continue to be chronically symptomatic in convalescence, including the elderly, those with multiple comorbidities and the hyper immune-excitable. These groups are particularly susceptible to serious infection with protracted recovery. PBM therapy is likely to improve cellular energy and general health status, lung immune function, gut microbiome/immune status, brain function and reduce muscle fatigue. We have also shown that PBM readily reverses anosmia in participants with Parkinson’s disease (manuscript in preparation). In the short term PBM therapy could improve recovery from COVID-19 and reduce the risk of post-infection sequalae. In the longer term PBM therapy could improve the comorbidities that increase vulnerability to viral infection in these populations. It would also be important to identify those younger hyper-excitable individuals who are at greater risk of an over-reaction to the viral infection.
3. Individuals who have been adversely affected by the lockdown and social isolation strategies in that they have been unable to regularly exercise and/or attend their normal rehabilitation session are likely to also benefit greatly from PBM therapy in the same ways as detailed above.
4. An additional benefit of PBM is as an adjuvant to vaccination. The elderly and those with comorbidity are most prone to be non- or under-responders to vaccines [106]. Kashiwagi et al [107] demonstrated that near infrared laser acts as an adjuvant to vaccination and significantly increases immune responses to intradermal influenza vaccination without augmenting Immunoglobulin-E. This conferred increased protection compared to an unadjuvanted vaccine control in a mouse influenza lethal challenge model. Thus, it is an exciting hypothesis for PBM to act as a non-invasive, risk-free and easily deployed adjuvant therapy, especially for at-risk populations.

## Conclusions

COVID-19 is not only a major challenge in people with comorbidities that affect their immune and inflammatory status, but is also particularly aggressive in the elderly, who have the compounding problems of an aging immune response, increased baseline inflammation, increased mitochondrial dysfunction and decreasing microbial diversity in their gut microbiome. PBM therapy is worthy of further rapid evaluation and could offer a safe, non-invasive, side-effect free and easily deployed adjunctive treatment and prevention, particularly suited for the most at-risk populations. Studies to evaluate the role of PBM in combatting COVID-19 infection and disease prevention may ultimately not only benefit the elderly and chronically sick but could have larger ramifications as a low risk, low-cost intervention in the prevention, treatment and healing of a variety of conditions. This may have implications for the most vulnerable individuals impacted by COVID-19, especially the elderly, infected with the virus, those who are slow to recover from the effects of the infection and those who have been denied their normal exercise/rehabilitation programs due to the necessary isolation restrictions.
